# Adapting the Advanced Cardiac Life Support for the Experienced Provider (ACLS-EP) course for emergency care education in Rwanda

**DOI:** 10.4102/phcfm.v3i1.178

**Published:** 2011-03-03

**Authors:** William E. Cayley Jr

**Affiliations:** 1 Department of Family Medicine, University of Wisconsin

**Keywords:** emergency care, family medicine, primary care, resuscitation, Rwanda

## Abstract

The Advanced Cardiac Life Support for the Experienced Provider (ACLS-EP) course uses a case-based curriculum to teach emergency resuscitation principles to experienced health care professionals. This article describes the adaptation of the ACLS-EP curriculum to be used in a family medicine training programme in Rwanda, including lessons learned and recommendations for future use of this material for emergency care education in the African setting.

## Introduction

Emergency care of sick or injured patients is one of the many challenges facing front-line general practice and family medicine physicians in sub-Saharan Africa. Rural district hospitals are often staffed by general physicians with only internship-level training. Protocols may be available to guide emergency care, ranging from locally written guidelines to international programmes such as the World Health Organization (WHO) programmes for Integrated Management of Childhood Illness (IMCI)^1^ and Integrated Management for Emergency and Essential Surgical Care (IMEESC),^2^ but these do not always provide guidance for the advanced management of complicated patient presentations. Furthermore, international protocols may not be directly relevant to local conditions, or may assume a level of medical technology not available in rural African hospitals (as is the case with cardiopulmonary resuscitation guidelines from the American Heart Association)^3^ and the European Resuscitation Council.^4^ Finally, protocol-driven instruction may not facilitate the level of critical thinking needed to train doctors for management of complicated patient presentations.

The American Heart Association’s course in Advanced Cardiac Life Support for the Experienced Provider (ACLS-EP)^5,6^ uses a case-based curriculum to teach emergency resuscitation principles to experienced health care professionals. The format is structured to include basic coverage of standard resuscitation algorithms, as well as group discussion of each case and topic to encourage critical thinking and adaptation of the teaching topics to local needs, resources and clinical realities (see Box 1). The ACLS-EP instructor’s manual specifically states that the course material is designed to be flexible, and ‘modification is encouraged as long as key objectives are met’.^6^

**BOX 1:**
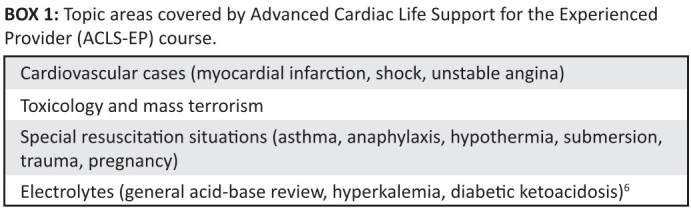
Topic areas covered by Advanced Cardiac Life Support for the Experienced Provider (ACLS-EP) course

Rwanda faces many of the health and medical challenges common to resource-poor countries in Africa: much of the population is rural, there is a very low ratio of physicians to population, and health care technology in rural areas is often very basic.^7^ In line with Rwanda’s vision 2020 health goals, the National University of Rwanda has developed a programme in Family and Community Medicine (FAMCO), both to help raise the level of primary care training in Rwanda and to improve the supply of primary care physicians.^8,9^ While trainees in this programme have already completed a two-year district hospital posting, familiarity with some aspects of basic resuscitation and emergency care is limited, even at this stage of training.

This paper discusses the use of the ACLS-EP curriculum in the Rwandan FAMCO primary care training programme, addressing strengths and weaknesses of the ACLS-EP materials for this setting and lessons that were learned, to inform future use of ACLS-EP material in similar settings.

## Method

As part of the FAMCO postgraduate training, an emergency medicine course was presented in November 2009. The ACLS-EP lecture slides and lecture notes were used to organise topic presentations and discussions (see Box 2).

**BOX 2:**
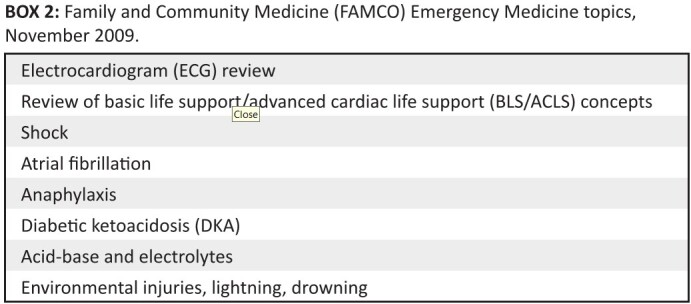
Family and Community Medicine (FAMCO) Emergency Medicine topics, November 2009

Four steps were taken to enhance the relevance of the presentations to the participating FAMCO trainees:

1. Topic content and case descriptions were edited on the basis of the author’s prior experience with the practice and teaching of emergency care in East Africa.
2. During case discussions, the trainees were asked to discuss the ways in which emergency care cases could be managed, both under optimal conditions in the country’s advanced hospitals, and under more resource-limited settings in the district hospitals.
3. The trainees were regularly asked to consider what sources of information could be used in each case to guide adaptation of emergency care recommendations to resource-limited district hospitals.
4. The trainees were also asked to reflect on the ways in which adaptation of emergency care recommendations to resource-limited settings could shed light on essential versus less essential aspects of care (for example, ‘How does managing diabetic ketoacidosis [DKA] with minimal laboratory availability help us to think about what is truly needed versus not needed to provide safe management of DKA?’)

The course was evaluated through discussions with both learners and colleagues, specifically with attention to the suitability of the curriculum as adapted for teaching in the Rwandan context, and learner reaction to the case-based teaching method.

## Results

Due to time constraints, not all of the cases in each ACLS-EP topic were covered. However, at the end of the one-week course, several themes regarding the use of the ACLS-EP curriculum for primary care training in emergency medicine in Rwanda emerged from the discussions with trainees and colleagues:

1. Many of the ACLS-EP cases are written assuming that there is a pre-hospital emergency medical services (EMS) system, and a means for activating it (e.g. a 9-1-1 telephone call). In addition, some of the cases assume availability of medications or medical technology that is not uniformly available in rural settings in developing countries. Thus, adaptation of the teaching material and cases to local realities is necessary.
2. Some topics were less applicable to the Rwandan setting (e.g. hypothermia) and some cases also used narratives that were less applicable to routine rural care in Rwanda (e.g. a lightning-strike case set on a golf course).
3. Use of the case discussion format engaged the learners in active discussion of how to adapt guideline-based recommendations to local realities, both safely and using sound medical reasoning. These discussions dealt both with how to make appropriate medical decisions regarding the case at hand when not all the needed medical information is available (e.g. a lack of laboratory results), and how to address system issues in order to make the limited resources that might be present more accessible.
4. Similarly, discussions regarding application of resuscitation guidelines in specific cases, when not all recommended tests or interventions would be available, led to debate and discussion amongst learners as to which tests, procedures, or practices are medically essential for good outcomes, and which are not.
5. Case discussions led to identification of systems issues that, if addressed, could facilitate improved care. For example, discussion of cardiac arrest scenarios led the learners to discuss and strategise ways to work with local hospital decision-makers to facilitate easier access to defibrillators, which, in some cases, were described as usually kept under lock and key and therefore not sufficiently available for emergency care.
6. The basic format of the ACLS-EP curriculum worked well for this teaching setting. The case materials include sufficient background information to support the teacher in guiding discussion, and the curriculum is readily available, yet teaching slides also allow for editing to allow adaptation of the material to the specific needs and realities of a given course.

## Discussion

The 2001 Utstein Symposium on Education in Resuscitation made several specific recommendations for the education of health care professionals in basic and advanced resuscitation skills (see Box 3).^10^ This course adapted ACLS-EP training to the Rwandan context in line with several aspects of the Utstein recommendations, including using ‘scenario-based, facilitated, interactive teaching’, extending content to ‘take into account specific emergencies that participants are likely to encounter’, and training in pre-arrest conditions. The course both demonstrated the feasibility of using the ACLS-EP curriculum for emergency medicine education in the Rwandan setting, and identified specific areas needed for appropriate adaptation to the local setting. In addition, this course demonstrated the value of case-based discussions for engaging learners in critical discussion and debate regarding medical management and problem solving to address local systems issues that impede care.

**BOX 3:**
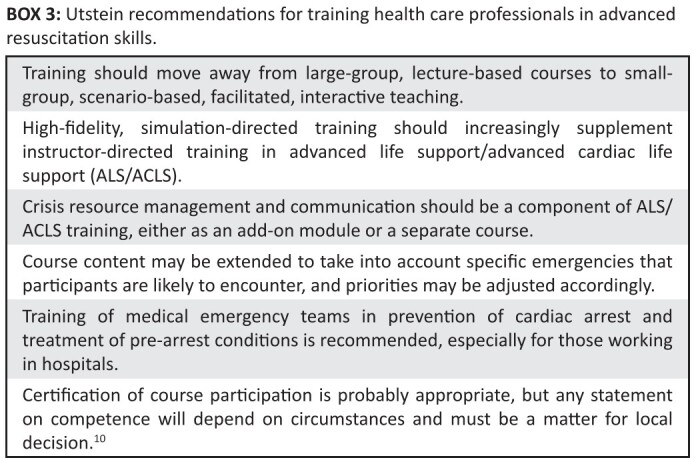
Utstein recommendations for training health care professionals in advanced resuscitation skills

## Limitations of the study

Limitations of this study include the fact that the course focused on cognitive training only, and did not include simulation or practice of clinical skills. In addition, since this is a case report of one educational intervention, and since the small size of the intervention did not allow for comparisons between two or more types of educational strategies, conclusions regarding optimal methods for course adaptation are difficult to make.

## Conclusion

While multiple resources and curricula are available for training in emergency care, the realities of general practice in sub-Saharan Africa, especially in rural settings, mean that many curricula do not directly address the day-to-day clinical challenges facing many African general practitioners. The ACLS-EP course provides one curriculum that is already designed to encourage the small-group, scenario-based, interactive teaching advocated by the Utstein recommendations, and also is in a format that can be adapted to the African setting with ease. The successful use of the course, however, will require the educator’s careful attention to that adaptation, to ensure relevance and appropriateness.
